# Supplementary material for: MetaRibo-Seq measures translation in microbiomes
Source: Nat Commun. 2020 Jun 29;11:3268. doi: 10.1038/s41467-020-17081-z (PMC7324362; doi:10.1038/s41467-020-17081-z)
Supplement: Supplementary file 10 — Supplementary Data 7 [file 41467_2020_17081_MOESM10_ESM.zip › File2/Confidence_VeryHigh_Taxonomy/89827_out.krona.html]

Javascript must be enabled to view this page.

members
magnitude
magnitudeUnassigned
count
unassigned
taxon
rank

89827\_out

4

superkingdom
2
4

phylum
3
1239

class
1
186801

order
1
186802

family
541000
1

1
216851
genus

1
853
species

SRS019068\_contig\_number\_98160

class
91061
2

186826
2
order

family
1300
2

2
1301
genus

species
712633
1

SRS148724\_contig\_number\_contig-100\_21796.60480

1
1759399
species

SRS102808\_contig\_number\_13956

phylum
1
1224

28216
1
class

order
206351
1

family
481
1

genus
482
1


SRS018822\_contig\_number\_contig-100\_3596.39742
1715205
1
species
